# Depression mediates motor dysfunction’s effect on sleep quality in ALS: a mediation analysis study

**DOI:** 10.3389/fnins.2025.1643367

**Published:** 2026-01-12

**Authors:** Yuchen Zhu, Gan Zhang, Hong Liu, Tielun Yin, Jiaying Zhang, Yanjing Yang, Linna Bai, Xiaoxuan Liu, Dongsheng Fan, Shan Ye

**Affiliations:** 1 Department of Neurology, Peking University Third Hospital, Beijing, China; 2 Beijing Key Laboratory of Biomarker and Translational Research in Neurodegenerative Diseases, Beijing, China; 3 Key Laboratory for Neuroscience, National Health Commission/Ministry of Education, Peking University, Beijing, China

**Keywords:** amyotrophic lateral sclerosis, depression, mediation analysis, motor dysfunction, sleep quality

## Abstract

**Introduction:**

Poor sleep quality affects 50–63% of Amyotrophic lateral sclerosis (ALS) patients, exacerbating disease burden and reducing quality of life. This study aimed to investigate the relationships among disease severity, depressive symptoms, and sleep quality in ALS, with a focus on the mediating effects of depression.

**Methods:**

Our study enrolled 408 ALS patients. Disease severity was assessed using the ALS Functional Rating Scale-Revised (ALSFRS-R), sleep quality via the Pittsburgh Sleep Quality Index (PSQI), and psychological status using the Hamilton Depression (HAMD) and Anxiety (HAMA) scales. Statistical analyses included Spearman correlations, multivariate regression, and mediation analysis (Hayes’ PROCESS macro).

**Results:**

Poor sleep quality (PSQI > 5) was observed in 54.4% of patients. Multivariate analysis found ALSFRS-R (*β* = −0.135, *p* = 0.042) and HAMD (*β* = 0.270, *p* < 0.001) correlated with sleep quality. Initial mediation analysis using the full ALSFRS-R and PSQI scales was not significant. Aimed to further explore the correlation, we derived specific subscales ALSFRS-R’ (motor/respiratory components) and PSQI’ (sleep efficiency/ daytime dysfunction), which more correlated with each other. Mediation analysis of these subscales revealed that depressive symptoms accounted for 36.3% of the indirect effect between ALSFRS-R’ and PSQI’.

**Discussion:**

Our cross-sectional exploratory study suggests that depression may partly mediate the relationship between motor dysfunction and poor sleep quality in patients with ALS. Although our mediation analysis suggested a potential association, further longitudinal cohort studies are needed to confirm these findings. The potential mediating role of depression underscores the need for an integrated clinical management approach addressing not only motor symptoms but psychological well-being as well.

## 1 Introduction

Amyotrophic lateral sclerosis (ALS) is a progressive and fatal neurodegenerative disorder of the central nervous system, clinically characterized by progressive muscle weakness and concomitant muscle atrophy (Hardiman et al., 2017; Feldman et al., 2022). Notably, non-motor symptoms may emerge as early as the initial disease stages, including cognitive and behavioral alterations, sleep disorders, pain, and autonomic dysfunction. Given the current lack of disease-modifying therapies to halt motor symptom progression, improving life quality is important in ALS management strategies (Mercadante and Al-Husinat, 2023).

Cross-sectional studies indicate that poor sleep quality affects 50 to 63% of ALS patients, as defined by the Pittsburgh Sleep Quality Index (PSQI > 5). Poor sleep quality may be related to emotional disturbances in ALS patients. On the other hand, it may also result from the degeneration of motor neurons, which leads to muscle weakness, stiffness, and sleep-related breathing disorders. Within the biopsychosocial framework, progressive physical disability in ALS may lead to psychological distress, including depression, which in turn can exacerbate sleep disturbances through mechanisms such as hyperarousal, rumination, and altered circadian rhythms. Depression is known to directly affect sleep architecture and perception of sleep quality, making it a plausible mediator in the pathway from motor impairment to poor sleep. The potential interrelationship among the three factors may be highly significant, which may offer insights into the formulation of therapeutic strategies for patients with ALS.

The study aims to investigate the relationship between ALS-related motor symptoms, emotional disturbances, and poor sleep quality. We hypothesize that motor dysfunction may be associated with decreased sleep quality in ALS patients, potentially partly mediated by emotional disturbances.

## 2 Materials and methods

### 2.1 Participates

From September 2022 to May 2024, we consecutively recruited patients with ALS hospitalized in the Department of Neurology at the Capital International Airport Campus of Peking University Third Hospital, Beijing, China. Patients were diagnosed with definite, probable or laboratory-supported probable ALS according to the revised El-Escorial criteria of the World Federation of Neurology. All participants were over 18 years old. Patients who refused to participate or had other neurological diseases that could interfere with the evaluation were excluded.

This study was approved by the Peking University Third Hospital Medical Science Research Ethics Committee (M2022511), and informed consent was obtained from all participants.

### 2.2 Clinical screening

We collected demographic and clinical data for all patients, including age, sex, education, body mass index (BMI), site of symptom onset, disease duration, bulbar involvement, use of non-invasive ventilation (NIV) and forced vital capacity (FVC). Disease severity was evaluated using the Amyotrophic Lateral Sclerosis Functional Rating Scale-Revised (ALSFRS-R). In this study, the progression rate (ΔALSFRS-R) was also calculated for patients with ALS. Consistent with prior studies, ΔALSFRS-R was defined as (48 − ALSFRS-R scores) / disease duration. Sleep quality was assessed using the Pittsburgh Sleep Quality Index (PSQI), with a PSQI score > 5 indicating poor sleep quality (Morin et al., 2011). Depression and anxiety were evaluated using the Hamilton Depression Scale (HAMD) and Hamilton Anxiety Scale (HAMA), respectively. Cognitive was assessed using Edinburgh cognitive and behavioural ALS screen (ECAS). Clinical staging was determined using the King’s clinical staging system (KCSS), and patients with ALS were assigned to their corresponding King’s clinical stages by at least two trained neurologists. The clinical and demographic results are presented in Table 1. All missing values were handled using the pairwise deletion method.

**Table 1:** Clinical characteristic of patients with ALS.

Characteristic	Patients with ALS
*N*	408
Age, y, median ± IQR	54.000 ± 14.400
Male, *n*, %	244 (59.804%)
BMI, mean ± SD	24.006 ± 3.095
Education, y, median ± IQR	12.000 ± 7.000
Bulbar onset, *n*, %	58 (14.216%)
Disease Duration, m, median ± IQR	14.000 ± 13.000
Bulbar involvement, *n*, %	147 (36.118%)
Use of NIV, *n*, %	17 (4.709%)
ALSFRS-R, median ± IQR	42.000 ± 6.000
ΔALSFRS-R, median ± IQR	0.425 ± 0.512
KCSS, *n*, %	
1	147 (40.720%)
2	140 (38.781%)
3	56 (15.512%)
4	18 (4.986%)
FVC, %, mean ± SD	89.989 ± 15.759
PSQI, median ± IQR	6.000 ± 5.000
HAMA, median ± IQR	3.000 ± 7.000
HAMD, median ± IQR	6.000 ± 9.000
ECAS, median ± IQR	97.000 ± 29.000

ALS, Amyotrophic lateral sclerosis; BMI, body mass index; NIV, non-invasive ventilation; ALSFRS-R, Amyotrophic Lateral Sclerosis Functional Rating Scale-Revised; KCSS, King’s clinical staging system; FVC, forced vital capacity; PSQI, Pittsburgh Sleep Quality Index; HAMA, Hamilton Anxiety Scale; HAMD, Hamilton Depression Scale; ECAS, Edinburgh cognitive and behavioural ALS screen.

### 2.3 Statistical analysis

We conducted the statistical analysis using a commercially available software package (SPSS, version 27.0; IBM). Based on data characteristics and distribution, Mann–Whitney U tests were used for pairwise comparison, and the Kruskal-Wallis test with Bonferroni correction was applied for multiple comparisons. Correlation analyses were performed using Spearman’s correlation and multiple linear regression analysis. Residuals from the multiple linear regression models were examined for normality using visual inspection of Q-Q plots, confirming no substantial deviations from normality that would violate model assumptions. Multicollinearity was evaluated using variance inflation factors (VIF) and tolerance statistics. All VIF values were below 5.0 (tolerance > 0.2), indicating no concerning multicollinearity among predictors in the final multivariate model. To further explore the underlying mechanism of the significant negative impact of motor dysfunction on sleep quality, depression was introduced as a mediating variable in the structural equation model. The mediating effect was examined using Model4 in the SPSS macro program Process, and the mediating role of depression in the relationship between motor dysfunction and sleep quality was verified and analyzed according to the Bootstrap method proposed by Hayes. Bootstrapping with 5,000 resamples was applied to estimate indirect effects and generate bias-corrected 95% confidence intervals. A *p*-value < 0.05 was considered significant in all analyses.

## 3 Results

### 3.1 Decreased sleep quality in patients with ALS

**Figure 1:**
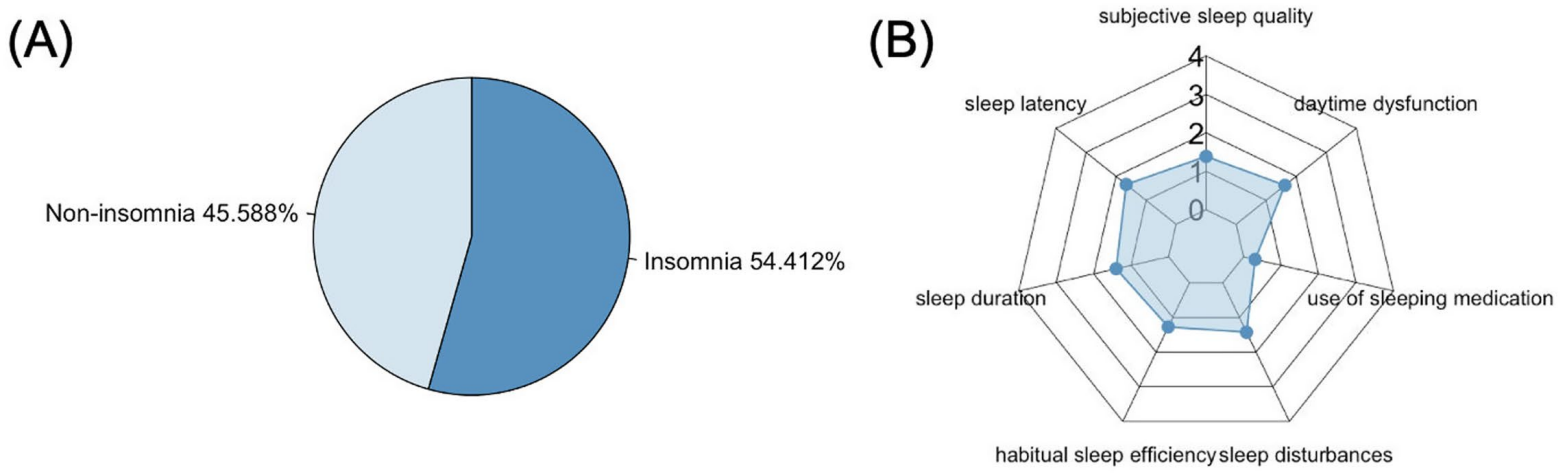
Decreased sleep quality in patients with ALS. **(A)** Proportion of ALS patients with poor sleep quality (PSQI > 5). **(B)** Radar chart showing the mean scores of the seven components of the PQSI in ALS patients.

In our study, 54.4% of patients with ALS had poor sleep quality (PSQI > 5) (Figure 1A) (Morin et al., 2011). The mean score of the seven components were presented in the radar chart (Figure 1B).

### 3.2 Sleep quality correlated with motor dysfunction and depression

**Figure 2:**
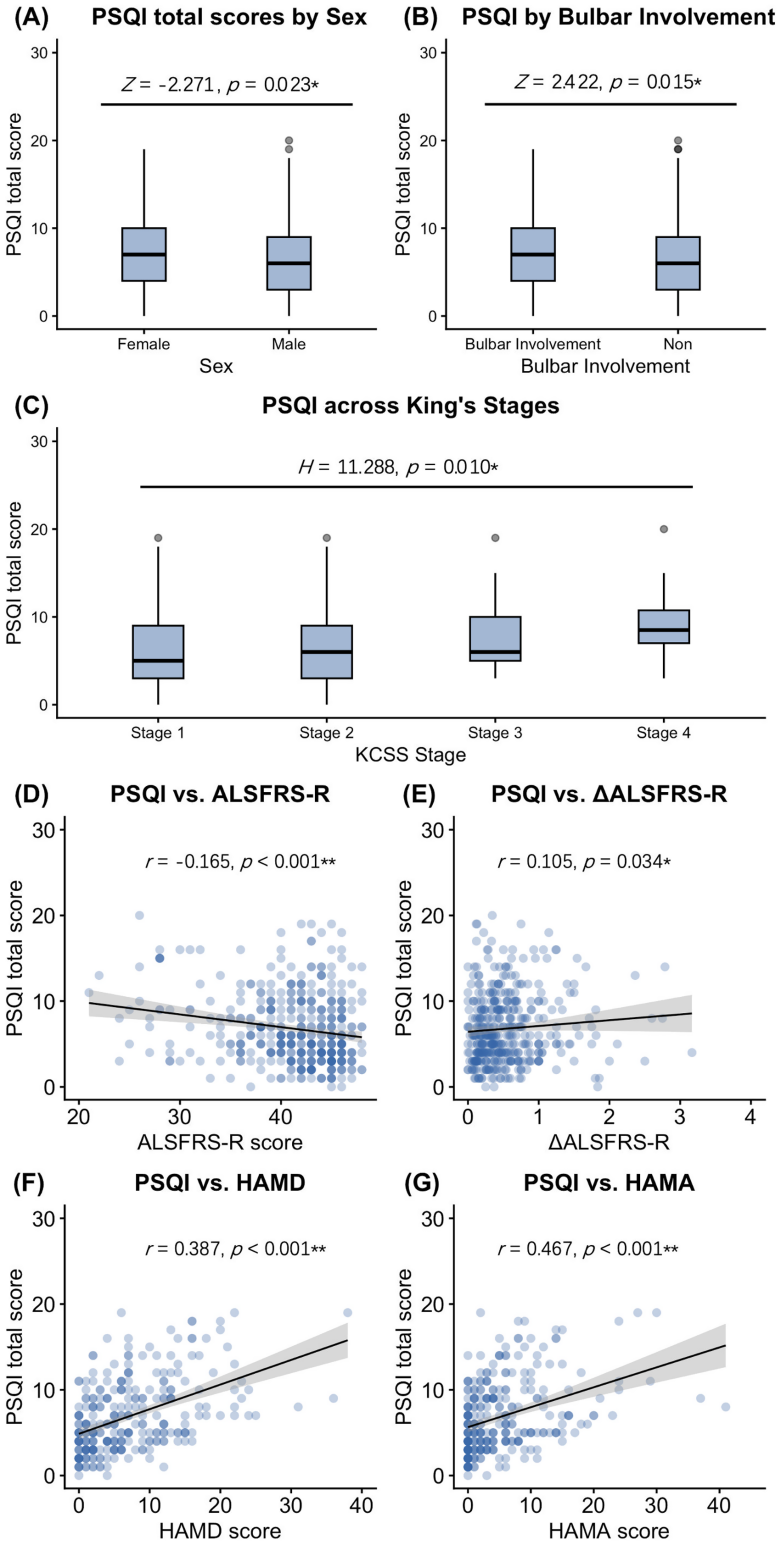
Associations between clinical characteristics and PSQI scores in ALS patients. **(A)** PSQI scores were significantly higher in females compared to males (*Z* = −2.271, *p* = 0.023). **(B)** Patients with bulbar involvement exhibited significantly higher PSQI scores (*Z* = 2.422, *p* = 0.015). **(C)** PSQI scores differed significantly across KCCS stages (*H* = 11.288, *p* = 0.010). **(D)** PSQI was negatively correlated with ALSFRS-R (*r* = −0.165, *p* < 0.001). **(E)** PSQI was positively correlated with ΔALSFR-RS (*r* = 0.105, *p* = 0.034). **(F)** PSQI was positively correlated with HAMD (*r* = 0.387, *p* < 0.001). **(G)** PSQI was positively correlated with HAMA (*r* =0.467, *p* <0.001). The plots illustrate the 25th and 75th percentiles (boxes), adjacent values (whiskers), outliers (dots), and median values of the groups (black horizontal lines in boxes).

In the univariate analysis, the PSQI score was significantly higher in female patients (*Z* = −2.271, *p =* 0.023) and those with bulbar involvement (*Z* = 2.422, *p =* 0.015). PSQI was negatively correlated with ALSFRS-R (*r* = −0.165, *p* < 0.001) and positively correlated with ΔALSFRS-R (*r* = 0.105, *p* = 0.034), HAMA (*r* = 0.387, *p* < 0.001), and HAMD (*r* = 0.467, *p* < 0.001). Additionally, PSQI scores differed across different KCSS stages (*H* = 11.288, *p* = 0.010) (Table 2, Figure 2).

**Table 2:** PSQI scores and clinical examinations.

Variable	*r/Z*/*H*	*p*
Age^c^	0.090	0.068
Sex^a^	−2.271	0.023*
BMI^c^	−0.049	0.321
Edu^c^	−0.069	0.165
Bulbar Onset^a^	1.523	0.128
Disease Duration^c^	0.053	0.286
Bulbar Involvement^a^	2.422	0.015*
Use of NIV^a^	1.686	0.092
ALSFRS-R^c^	−0.165	<0.001**
ΔALSFRS-R^c^	0.105	0.034*
KCSS^b^	11.288	0.010*
FVC%^c^	−0.183	0.172
HAMA^c^	0.387	<0.001**
HAMD^c^	0.467	<0.001**
ECAS^c^	0.031	0.733

ALS, Amyotrophic lateral sclerosis; BMI, body mass index; NIV, non-invasive ventilation; ALSFRS-R, Amyotrophic Lateral Sclerosis Functional Rating Scale-Revised; KCSS, King’s clinical staging system; FVC, forced vital capacity; PSQI, Pittsburgh Sleep Quality Index; HAMA, Hamilton Anxiety Scale; HAMD, Hamilton Depression Scale; ECAS, Edinburgh cognitive and behavioural ALS screen.

a The Mann Whitney test was used for comparisons.

b The Kruskal–Wallis test was used for multiple comparisons.

c Spearman correlation was used for correlation analyze.

*p < 0.05; **p < 0.01.

In the multivariate analysis, we adjusted for all variables with *p* < 0.100 in the univariate analysis, including age, sex, bulbar involvement, use of NIV, ALSFRS-R, ΔALSFRS-R, KCSS, HAMA, and HAMD. We found that PSQI was negatively correlated with ALSFRS-R (*r =* −0.135, *p =* 0.042) and positively correlated with HAMD (*r =* 0.270, *p* < 0.001) (Table 3). Additionally, no multicollinearity was detected among the variables in the model.

**Table 3:** Multivariate analysis of PSQI.

Variable	*β*	*p*
Age	0.025	0.264
Sex	−0.532	0.278
Bulbar Involvement	0.406	0.505
Use of NIV	0.211	0.860
ALSFRS-R	−0.135	0.042*
ΔALSFRS-R	−0.451	0.462
KCSS	−0.207	0.610
HAMA	−0.001	0.985
HAMD	0.270	<0.001**

PSQI, Pittsburgh Sleep Quality Index; ALSFRS-R, Amyotrophic Lateral Sclerosis Functional Rating Scale-Revised; NIV, non-invasive ventilation; KCSS, King’s clinical staging system; HAMA, Hamilton Anxiety Scale; HAMD, Hamilton Depression Scale.

*p < 0.05; **p < 0.01.

### 3.3 Depression mediates the effect of motor dysfunction on sleep quality

**Figure 3:**
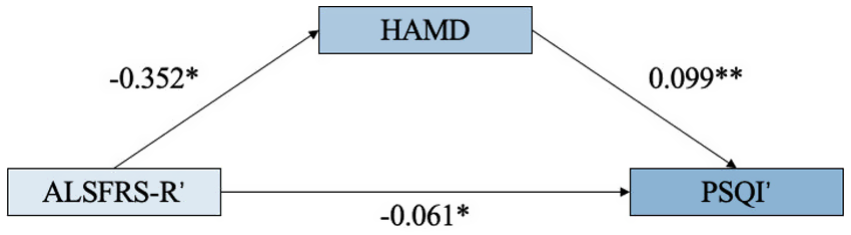
Path diagram of mediation analysis. ALSFRS-R’ (motor/respiratory components) had a direct effect on PSQI’ (sleep efficiency/daytime dysfunction) (β = −0.096, *p* =0.004) and an indirect effect mediated by HAMD (β = −0.035).

We further investigated whether depression may mediate the impact of motor dysfunction on sleep quality. The mediation analysis results were not statistically significant. This lack of significance may be attributable to varying impacts of specific ALSFRS-R components on sleep outcomes, where certain subdomains (e.g., handwriting) showed weaker associations with sleep quality compared to other functional measures. To address this, we defined ALSFRS-R’ as the sum of 7 components correlated with PSQI (*p* < 0.100) (Supplementary Table 1) and PSQI’ as the sum of 2 components correlated with ALSFRS-R (*p* < 0.100) (Supplementary Table 2). This threshold was chosen to inclusively capture potentially relevant items while minimizing the omission of clinically plausible components. These derived subscals were used only in secondary mediation models to explore more specific pathways. Our analysis revealed a significant total effect of motor disability on poor sleep quality (*β* = −0.096, 95% CI: −0.162 to −0.031, *p* = 0.004). The indirect effect through depression was estimated at β = −0.035 (95% CI: −0.064 to −0.009), accounting for 36.3% of the total effect. However, the direct effect remained significant (*β* = −0.061, 95% CI: −0.122 to −0.001, *p* = 0.047), indicating partial mediation (Figure 3; Supplementary Table 3).

## 4 Discussion

Our study demonstrated that over half of the ALS patients experience impaired sleep quality. Further analysis revealed significant correlations between PSQI scores and both ALSFRS-R and HAMD scores. Mediation analysis indicated that depressive symptoms, as assessed by HAMD, partially mediate the effect of ALSFRS-R-defined motor dysfunction on sleep quality deterioration.

### 4.1 Motor dysfunction and sleep quality in ALS

Our study found that 54.4% patients with ALS had poor sleep quality, defined as PSQI > 5. This is consistent previous studies reporting rates of 50–63% (Diaz-Abad et al., 2018; Panda et al., 2018; Lucia et al., 2021). After adjusting for potential confounders, multivariable analysis revealed a persistent negative correlation between PSQI and ALSFRS-R scores. This suggests that motor dysfunction is independently associated with decreased sleep quality in patients with ALS. Previous studies have yielded conflicting results regarding the relationship between sleep quality and motor dysfunction. Lo Coco et al. (2011) reported significant associations between PSQI and ALSFRS-R scores. Panda et al. (2018) reported significant associations between PSQI and ALSFRS scores, while Diaz et al. found no statistically significant correlation (Diaz-Abad et al., 2018). These inconsistencies may be partially attributable to small sample sizes (all ≤100 participants). In our larger cohort (*n* = 408), PSQI demonstrated a clinically meaningful association with ALSFRS-R.

The pathophysiological mechanisms underlying sleep disturbances in patients with ALS are multifactorial and likely stem from motor symptom progression. Primarily, respiratory muscle weakness can lead to abnormal breathing and gas exchange, resulting in poor sleep quality (Ahmed et al., 2016). Additionally, secondary to motor neuron degeneration, symptoms such as muscle cramps, pain, spasticity, immobilization, and restless legs syndrome are recognized contributors to decreased PSQI (Boentert, 2020). Therefore, poor sleep quality is prevalent among ALS patients and is closely linked to motor dysfunction.

### 4.2 Emotional factors in ALS

Previous studies have reported depression prevalence rates of 9–11% in patients with ALS, assessed using structured interviews based on DSM-IV criteria (Kurt et al., 2007; De Marchi et al., 2019). Patients with ALS are at an elevated risk of developing depression within the first year after diagnosis (Roos et al., 2016), with depressive symptom severity positively correlated with physical impairment (Kübler et al., 2005). Depression is almost invariably associated with sleep abnormalities (Yasugaki et al., 2025). Multiple studies have demonstrated a significant association between poor sleep quality and depressive symptoms in ALS patients (Lo Coco et al., 2011; Diaz-Abad et al., 2018; Panda et al., 2018). Negative or ruminative thoughts linked to depression may lead to hypervigilance and subsequent sleep disturbances (Urrila et al., 2015).

Although anxiety is widely recognized to be associated with depression, anxiety does not mediate sleep disturbances in our study. This may reflect different pathophysiological and psychosocial mechanisms substantially from those of depression. Chronic motor dysfunction perpetuates activation of the hypothalamic–pituitary–adrenal (HPA) axis, thereby disrupting neurobiological substrates subserving mood and sleep regulation. Functional loss predominantly elicits loss-oriented depressive responses rather than threat-based anxiety, which more profoundly perturbs sleep architecture. Moreover, erosion of social roles and self-worth directly precipitates depressive symptomatology, whose impact on sleep markedly exceeds that of anxiety, establishing depression as the principal pathway linking motor dysfunction to sleep disturbance.

### 4.3 Depression as a partial mediator

To explore the multifaceted impact of motor dysfunction on sleep quality, we examined whether this relationship is mediated by depressive symptoms. Our mediation analysis indicated that depressive symptoms may partially mediated the association between motor dysfunction and sleep quality, with 36.3% of the total effect being attributable to indirect pathways. However, it is important to note that the cross-sectional design of this study limits causal inference. Although associations were observed, causality cannot be established. Moreover, the relationships among these variables are likely complex and bidirectional. For instance, sleep disturbances may worsen depressive symptoms and potentially amplify the perception of motor disability. Future longitudinal studies are needed to disentangle the temporal sequence and confirm the directionality of these associations. In current clinical practice, the focus often remains predominantly on physical impairment rather than on emotional disturbances as the disease progresses. Thus, the management of depressive symptoms may represent a potential target for patient care, and timely interventions could be explored to improve sleep quality and enhance overall quality of life.

When analyzing the mediating role of HAMD in the relationship between ALSFRS-R and PSQI, we did not find significant positive results. This non-significant finding may reflect the domain-specific nature of the relationships under investigation. The comprehensive ALSFRS-R assesses heterogeneous motor functions, some of which (e.g., handwriting, fine dexterity) may have less direct relevance to sleep physiology compared to others (e.g., limb mobility for repositioning in bed, respiratory muscle function). Similarly, the global PSQI score integrates multiple sleep dimensions, not all of which may be equally sensitive to motor decline or mood. After extracting components closely correlated with another scale, we found that HAMD partially mediated the effect of ALSFRS-R’ on PSQI’, with the mediation effect accounting for 36.3% of the total effect. For the ALSFRS-R’, we included component related to lower limb motor and respiration, while for PSQI’, we selected sleep efficiency and daytime dysfunction components. Diaz-Abad et al. reported that sleep quality was more correlated with the patient’s ability to turn in bed and adjust bed clothes than with ALSFRS scores (Diaz-Abad et al., 2018). Therefore, when patients exhibit lower limb motor dysfunction and respiratory issues, greater emphasis might be placed on assessing and intervening in emotional well-being and sleep quality in their care regimen.

### 4.4 Limitations and future direction

Our work highlights depression as a potential modifiable target for improving sleep quality and overall quality of life in ALS patients. These results align with growing recognition of the biopsychosocial model in neurodegenerative diseases and could inform guidelines for multidisciplinary care. There were several limitations. First, the cross-sectional design of our study precludes the establishment of causal relationships among motor dysfunction, depression, and sleep quality. Although mediation analysis suggests a potential pathway wherein depression partially mediates the effect of motor impairment on sleep disturbances, the temporal sequence of these variables cannot be definitively determined without longitudinal data. Future prospective studies are warranted to validate the mediating role of depression and to explore dynamic changes in these associations over the disease course. Second, only 4.17% of patients in this cohort used NIV, a rate notably lower than that typically reported in ALS populations, which may be attributed to the predominance of newly diagnosed patients in our cohort. Patients using NIV often present with more severe disease, and respiratory impairment in these individuals is a significant factor affecting sleep quality. Thus, low usage in our study may lead to an underestimation of the relationship between respiratory dysfunction and poor sleep quality. Third, we did not collect information on patients’ use of sedative-hypnotics, antidepressants, or related medications. Sedative-hypnotics may improve subjective sleep quality independent of the underlying disease or psychological state, while various antidepressants can differentially affect sleep architecture and reported depressive symptoms. Due to the potential confounding effects of medication use which we were unable to control for, the observed associations between motor dysfunction, depressive symptoms, and sleep quality, as well as the estimated mediation effect of depression, may have been influenced. Fourth, as a single-center study in China, cultural factors (e.g., stigma around mood disorders, sleep habits) may influence depression reporting and sleep perceptions. This may limit generalizability to non-Chinese populations. Finally, the mediation analysis using data-driven subscales (ALSFRS-R’ and PSQI’) was exploratory. We referenced the approach for selecting potential independent variables in multiple linear regression, adopting *p* < 0.1 as the threshold for constructing the PSQI’ and ALSFRSR’ scales, which may increase the risk of Type I error. While it suggests a potential pathway, these findings require validation in prospective studies using predefined subdomains.

Sleep quality impairment associated with depressive symptoms and motor dysfunction, and depressive symptoms may mediate the relationship between motor dysfunction and decreased sleep quality. Targeting depressive symptoms through pharmacological or behavioral interventions may help break the vicious cycle between ALS progression and sleep disorders. However, this is an exploratory study, and more in-depth research is warranted in the future.

## Data Availability

The raw data supporting the conclusions of this article will be made available by the authors, without undue reservation.

## References

[ref1] Ahmed R. M. Newcombe R. E. Piper A. J. Lewis S. J. Yee B. J. Kiernan M. C. . (2016). Sleep disorders and respiratory function in amyotrophic lateral sclerosis. Sleep Med. Rev. 26, 33–42. doi: 10.1016/j.smrv.2015.05.007, 26166297

[ref2] Boentert M. (2020). Sleep and sleep disruption in amyotrophic lateral sclerosis. Curr. Neurol. Neurosci. Rep. 20:25. doi: 10.1007/s11910-020-01047-1, 32462331 PMC7253511

[ref3] De Marchi F. Sarnelli M. F. Solara V. Bersano E. Cantello R. Mazzini L. (2019). Depression and risk of cognitive dysfunctions in amyotrophic lateral sclerosis. Acta Neurol. Scand. 139, 438–445. doi: 10.1111/ane.13073, 30712314

[ref4] Diaz-Abad M. Buczyner J. R. Venza B. R. Scharf S. M. Kwan J. Y. Lubinski B. . (2018). Poor sleep quality in patients with amyotrophic lateral sclerosis at the time of diagnosis. J. Clin. Neuromuscul. Dis. 20, 60–68. doi: 10.1097/cnd.0000000000000234, 30439751

[ref5] Feldman E. L. Goutman S. A. Petri S. Mazzini L. Savelieff M. G. Shaw P. J. . (2022). Amyotrophic lateral sclerosis. Lancet 400, 1363–1380. doi: 10.1016/s0140-6736(22)01272-7, 36116464 PMC10089700

[ref6] Hardiman O. Al-Chalabi A. Chio A. Corr E. M. Logroscino G. Robberecht W. . (2017). Amyotrophic lateral sclerosis. Nat. Rev. Dis. Primers 3:17071. doi: 10.1038/nrdp.2017.71, 28980624

[ref7] Kübler A. Winter S. Ludolph A. C. Hautzinger M. Birbaumer N. (2005). Severity of depressive symptoms and quality of life in patients with amyotrophic lateral sclerosis. Neurorehabil. Neural Repair 19, 182–193. doi: 10.1177/1545968305276583, 16093409

[ref8] Kurt A. Nijboer F. Matuz T. Kübler A. (2007). Depression and anxiety in individuals with amyotrophic lateral sclerosis: epidemiology and management. CNS Drugs 21, 279–291. doi: 10.2165/00023210-200721040-00003, 17381183

[ref9] Lo Coco D. Mattaliano P. Spataro R. Mattaliano A. La Bella V. (2011). Sleep-wake disturbances in patients with amyotrophic lateral sclerosis. J. Neurol. Neurosurg. Psychiatry 82, 839–842. doi: 10.1136/jnnp.2010.228007, 21217159

[ref10] Lucia D. McCombe P. A. Henderson R. D. Ngo S. T. (2021). Disorders of sleep and wakefulness in amyotrophic lateral sclerosis (ALS): a systematic review. Amyotroph. Lateral Scler. Frontotemporal Degener. 22, 161–169. doi: 10.1080/21678421.2020.1844755, 33191797

[ref11] Mercadante S. Al-Husinat L. (2023). Palliative care in amyotrophic lateral sclerosis. J. Pain Symptom Manag. 66, e485–e499. doi: 10.1016/j.jpainsymman.2023.06.029, 37380145

[ref12] Morin C. M. Belleville G. Bélanger L. Ivers H. (2011). The insomnia severity index: psychometric indicators to detect insomnia cases and evaluate treatment response. Sleep 34, 601–608. doi: 10.1093/sleep/34.5.601, 21532953 PMC3079939

[ref13] Panda S. Gourie-Devi M. Sharma A. (2018). Sleep disorders in amyotrophic lateral sclerosis: a questionnaire-based study from India. Neurol. India 66, 700–708. doi: 10.4103/0028-3886.232327, 29766929

[ref14] Roos E. Mariosa D. Ingre C. Lundholm C. Wirdefeldt K. Roos P. M. . (2016). Depression in amyotrophic lateral sclerosis. Neurology 86, 2271–2277. doi: 10.1212/wnl.0000000000002671, 27164661 PMC4909561

[ref15] Urrila A. S. Paunio T. Palomäki E. Marttunen M. (2015). Sleep in adolescent depression: physiological perspectives. Acta Physiol (Oxf.) 213, 758–777. doi: 10.1111/apha.12449, 25561272

[ref16] Yasugaki S. Okamura H. Kaneko A. Hayashi Y. (2025). Bidirectional relationship between sleep and depression. Neurosci. Res. 211, 57–64. doi: 10.1016/j.neures.2023.04.006, 37116584

