# Supplementary material for: Depression mediates motor dysfunction’s effect on sleep quality in ALS: a mediation analysis study
Source: Front Neurosci. 2026 Jan 12;19:1643367. doi: 10.3389/fnins.2025.1643367 (PMC12832777; doi:10.3389/fnins.2025.1643367)
Supplement: Supplementary file 1 [file Table_1.docx]

Supplementary Material

# Supplementary Table

## Supplementary Table 1 Correlations between PSQI component and ALSFRS-R score

| Variable | *r* | *p* |
| --- | --- | --- |
| PSQI | -0.165 | <0.001** |
| subjective sleep quality | -0.073 | 0.143 |
| sleep latency | -0.079 | 0.113 |
| sleep duration | -0.051 | 0.309 |
| habitual sleep efficiency | -0.184 | <0.001** |
| sleep disturbances | -0.073 | 0.144 |
| use of sleeping medication | -0.069 | 0.163 |
| daytime dysfunction | -0.150 | 0.003** |

Abbreviations: ALSFRS-R = Amyotrophic Lateral Sclerosis Functional Rating Scale-Revised; PSQI = Pittsburgh Sleep Quality Index;

*p < 0.05; **p < 0.01.

## Supplementary Table 2 Correlations between ALSFRS-R components and PSQI score

| Variable | *r* | *p* |
| --- | --- | --- |
| ALSFRS-R | -0.165 | <0.001** |
| Speech | -0.096 | 0.053 |
| Salivation | -0.031 | 0.532 |
| Swallowing | -0.077 | 0.119 |
| Handwriting | -0.064 | 0.199 |
| Cutting food and handling utensils | -0.072 | 0.148 |
| Dressing and hygiene | -0.062 | 0.209 |
| Turning in bed and adjusting bed clothes | -0.154 | 0.002** |
| Walking | -0.114 | 0.021* |
| Climbing stairs | -0.131 | 0.008** |
| Dyspnea | -0.118* | 0.017* |
| Orthopnea | -0.145* | 0.003** |
| Respiratory insufficiency | -0.096 | 0.053 |

Abbreviations: ALSFRS-R = Amyotrophic Lateral Sclerosis Functional Rating Scale-Revised; PSQI = Pittsburgh Sleep Quality Index;

*p < 0.05; **p < 0.01.

## Supplementary Table 3. Direct, Indirect, and Total Effects of ALSFRS-R’ on PSQI’ Through HAMD

| **Effect** | ***β*** | **SE** | **LLCI** | **ULCI** | ***p*** |
| --- | --- | --- | --- | --- | --- |
| **Total effect** | -0.096 | 0.03 | -0.162 | -0.031 | 0.004** |
| **Direct effect** | -0.061 | 0.03 | -0.122 | -0.001 | 0.047* |
| **Indirect effect** | -0.035 | 0.01 | -0.065 | -0.010 | - |

Abbreviations: ALSFRS-R = Amyotrophic Lateral Sclerosis Functional Rating Scale-Revised; PSQI = Pittsburgh Sleep Quality Index;

*p < 0.05; **p < 0.01.
